# Items

**Published:** 1894-10

**Authors:** 


### ITEMS.

Dr. Salmon, Chief of the Bureau of Animal Industry, has just ordered a return to Ireland of an importation of Shropshire sheep, landed at the Port of Baltimore, suffering from foot and mouth disease.

The Des Moines, Iowa, Veterinary College has closed its doors for lack of support. The day of two year schools, imperfectly equipped, has gone in this country, never to return.

The English reading veterinary world will soon have an extremely valuable contribution to their literature, in the translation, by Prof. W. L. Zuill, of Philadelphia, of the German work of Messrs. Friedberger and Fröhner, with the notes of the French translators and selections from those of Prof Trasbot.

McKillip Veterinary College, of Chicago, with a three years course, starts her initial year with sixteen matriculants, all of whom have passed the necessary entrance examination, as adopted by the Association of Veterinary Faculties of North America. This is a high compliment for a new school of advanced curriculum, and speaks well for the future profession in America.

Had all the existing and prospective veterinary colleges commenced their courses in North America this year, we would have had twenty veterinary schools to furnish new recruits to the ranks. Surely more than there could be any need of!

If the United States Veterinary Medical Association, in her thirty-one years of existence, has not many laurels to wear, she has one to adorn her crown that will shine long to her honor and glory—her persistent agitation and demand for a higher standard of veterinary education. Let the good work go on.

In at least one of the Western veterinary colleges, with a two years course of six months each, there is a great falling off in the matriculants for the year. Will not all the schools soon recognize that they must broaden their course or suffer materially in the growth of and respect for their schools?

Veterinarians of Baltimore, and many of the members of the United States Veterinary Medical Association, were willing contributors to a fund presented to the widow of our late associate, Dr. C. B. Michener.

